# Evidence consistency analysis of the effect of a low-FODMAP diet on the abundance of *Bifidobacterium* and *Faecalibacterium prausnitzii* in patients with irritable bowel syndrome

**DOI:** 10.3389/fmicb.2026.1948754

**Published:** 2026-10-06

**Authors:** Bingqian Cao, Li Zhou, Shouzhang Hu, Yue Huang

**Affiliations:** 1 School of Public Health, Wuhan University of Science and Technology, Wuhan, China; 2 Medical Imaging Department, Wuhan University of Science and Technology Hospital, Wuhan, China; 3 Institute of Food Safety and Nutrition Hygiene, Wuhan Center for Disease Control and Prevention, Wuhan, China

**Keywords:** *Bifidobacterium*, direction-of-effect and consistency analysis, *Faecalibacterium prausnitzii*, gut microbiota, irritable bowel syndrome, low FODMAP diet

## Abstract

**Background:**

The low-FODMAP diet is recommended by clinical guidelines as an effective dietary strategy for alleviating symptoms in patients with irritable bowel syndrome (IBS). However, its effect on *Bifidobacterium* and *Faecalibacterium prausnitzii* (*F. prausnitzii*) remains unclear, largely due to methodological heterogeneity.

**Methods:**

We systematically searched the literature up to September 2026 for randomized controlled trials comparing low-FODMAP diets with control diets in adults with IBS. Given the high methodological heterogeneity across studies, we performed a direction-of-effect and consistency analysis (vote counting and sign test) instead of a traditional meta-analysis.

**Results:**

12 RCTs were included. For *Bifidobacterium* (11 studies), 9 (81.8%) reported a decrease and 2 reported no change; the sign test confirmed a consistent reduction (*p* = 0.033). A sensitivity analysis including only studies with direct between-group comparisons (9 studies) was consistent with the aforementioned analysis (8 reported a decrease, *p* = 0.020). For *F. prausnitzii* (7 studies), the evidence was inconsistent: 2 reported a decrease, 4 reported no change, and 1 reported an increase, with no statistically significant trend.

**Conclusion:**

Most current evidence indicates that a low FODMAP diet reduces the abundance of *Bifidobacterium* in IBS patients, while its effect on *F. prausnitzii* remains unclear. This finding enables clinicians to conduct more evidence-based counseling with patients, clarifying the specific effects (and non-effects) of the low-FODMAP diet on microbiota and facilitating informed decision-making regarding dietary management. Furthermore, future research should investigate the dynamics of microbiota during the reintroduction phase and consider patient microbiota subtypes to clarify the overall impact of the diet on the microbiota.

**Systematic review registration:**

This systematic review has been registered with PROSPERO (International Prospective Register of Systematic Reviews) under the registration number CRD420261351146. The publicly accessible URL is: https://www.crd.york.ac.uk/PROSPERO/.

## 1 Introduction

Irritable bowel syndrome (IBS) is a prevalent disorder of gut-brain interaction that substantially impairs quality of life and increases healthcare burden (Drossman and Hasler, 2016). Alterations in the gut microbiota are recognized as a key pathophysiological factor (Ford et al., 2020). Dietary management, particularly the low fermentable oligo-, di-, monosaccharides, and polyols (FODMAP) diet, has been recommended by clinical guidelines, including the American College of Gastroenterology (ACG) and the American Gastroenterological Association (AGA), as an effective dietary strategy for alleviating gastrointestinal symptoms (Lacy et al., 2021; Chey et al., 2022). By restricting highly fermentable substrates, the low FODMAP diet reduces luminal distension (Staudacher et al., 2017). However, since FODMAPs serve as primary metabolic substrates for many commensal bacteria, this restriction raises important questions regarding its ecological impact on the gut microbiota (Sun et al., 2019).

Of particular interest are *Bifidobacterium* and *Faecalibacterium prausnitzii* (*F. prausnitzii*), two taxa commonly reduced in IBS patients compared to healthy controls (Liu et al., 2017). *Bifidobacterium* contributes to gut physiology and immune regulation through short-chain fatty acid production (Bonfrate et al., 2020; Milani et al., 2015; Falony et al., 2006). In animal models of IBS, *Bifidobacterium. Longum* improved defecation habits, visceral hypersensitivity, and mucosal repair, partly via inhibition of the NLRP3 inflammasome (Zhou et al., 2020; Gu et al., 2016). Clinical studies have shown that supplementation with specific *Bifidobacterium* strains can improve IBS symptoms and abdominal discomfort (Martin et al., 2024; Andresen et al., 2020). Similarly, *F. prausnitzii* is a major butyrate producer and a biomarker of intestinal health (Miquel et al., 2016; Hustoft et al., 2016). Animal studies have demonstrated its anti-inflammatory effects and its ability to improve intestinal permeability and visceral perception in IBS and colitis models (Sokol et al., 2008; Miquel et al., 2015; Scharf et al., 2025). Given their association with IBS, clarifying how the low-FODMAP diet affects these two taxa is clinically important. Preliminary evidence suggests this diet may reduce their abundance, prompting concerns about the diet’s long-term safety and functional impacts (Staudacher et al., 2017; Liu et al., 2020; Vasant et al., 2021).

Previous research has drawn inconsistent conclusions about the effects of a low FODMAP diet on *Bifidobacterium* and *F. prausnitzii*. Some reported reductions in both taxa (Staudacher et al., 2017; Halmos et al., 2015; Staudacher et al., 2012), potentially attributable to their ability to degrade fructans, which are restricted in this diet (Pokusaeva et al., 2011). While others reported no significant changes or even improvements in microbial diversity (Chu et al., 2025). These conflicting results indicate that a comprehensive synthesis is needed (Chu et al., 2025). However, existing reviews have been constrained by a methodological challenge: the high heterogeneity across studies in microbiome collection, sequencing, preprocessing, and reporting makes traditional meta-analysis inappropriate (So et al., 2022; Willis, 2019). Consequently, whether the low FODMAP diet affects specific bacterial taxa remains unclear. Understanding this is crucial for developing personalized nutritional strategies for IBS patients (Quigley, 2013).

Therefore, to assess whether a consistent pattern exists, we conducted a systematic review using a vote counting based on direction of effect analysis. This method summarizes how many studies report an increase, decrease, or no change for a given outcome, which can be useful when studies are too heterogeneous for traditional meta-analysis (Page et al., 2021). The aim of this review was to assess the evidence for a consistent directional change in the abundance of *Bifidobacterium* and *F. prausnitzii* in adults with IBS following a low FODMAP diet.

## 2 Materials and methods

### 2.1 Study design and registration

This systematic review employed a vote counting method based on direction of effect and consistency analysis to synthesize evidence regarding a low FODMAP diet’s effects on the abundance of gut *Bifidobacterium* and *F. prausnitzii* in IBS patients. This approach is recommended by the Cochrane Handbook when meta-analysis is not feasible (Campbell et al., 2020), and has been successfully used in multiple systematic reviews to evaluate directional trends across different study designs (Boon and Thomson, 2021; Kardan et al., 2024). The reporting follows PRISMA. The publicly accessible URL of PROSPERO is: https://www.crd.york.ac.uk/PROSPERO/.

### 2.2 Search strategy

A systematic literature search was conducted in PubMed, Medline, the Cochrane Library, Google Scholar, and Web of Science Core Collection from inception to September 2026. The search strategy was built around three core concepts: “Irritable Bowel Syndrome,” “low-FODMAP diet,” and “gut microbiota.” The following was an example search strategy for PubMed: (“FODMAP”[Title/Abstract] OR “Fermentable oligo-, di-, mono-saccharides and polyols”[Title/Abstract]) AND (“Irritable Bowel Syndrome”[Mesh] OR “IBS”[Title/Abstract]) AND (“Gastrointestinal Microbiome”[Mesh] OR “Microbiota”[Title/Abstract] OR “Microbiome”[Title/Abstract] OR “*Bifidobacterium*”[Title/Abstract] OR “*Faecalibacterium*”[Title/Abstract]) Additionally, the reference lists of relevant reviews were manually screened to identify any potentially missed studies.

### 2.3 Eligibility criteria

The research question was structured according to the PICO (Population, Intervention, Comparison, Outcomes) framework, which also informed the development of our eligibility criteria. Study Design: Randomized controlled trials (RCTs), including both parallel-group and crossover designs. For crossover trials, data were extracted only from the first intervention period (pre-crossover) to avoid carryover effects. Participants: Adult patients (≥18 years) diagnosed with IBS according to any version of the Rome criteria (Drossman, 2016; Drossman and Dumitrascu, 2006), irrespective of subtype. Intervention: A comprehensive low-FODMAP diet involving the systematic restriction of all high-FODMAP food categories, delivered via dietary advice or controlled provision of meals. Comparator: Any controlled dietary regimen, including but not limited to a habitual diet, sham diet, high-FODMAP diet, or traditional dietary advice. Outcomes: Studies must report quantitative data on gut microbiota composition at both baseline and post-intervention (e.g., derived from 16S rRNA gene sequencing, qPCR, or shotgun metagenomics), allowing for the extraction of data on the abundance of *Bifidobacterium* and *F. prausnitzii*. Exclusion Criteria: Non-randomized studies, single-arm trials, studies involving non-IBS populations (e.g., inflammatory bowel disease, pediatric patients), studies testing isolated FODMAP components, studies combining the low-FODMAP diet with a co-intervention (e.g., probiotics) where the dietary effect could not be isolated, studies not reporting data on the target bacterial taxa, and non-original research (e.g., reviews, meta-analyses).

### 2.4 Study selection and data extraction

Study selection was performed independently by two reviewers. Initial screening was based on titles and abstracts, followed by full-text assessment of potentially eligible studies. Any discrepancies were resolved through discussion or by consulting a third reviewer. The selection process was documented using a PRISMA (Liberati et al., 2009) flow diagram (Figure 1).

**Figure 1:**
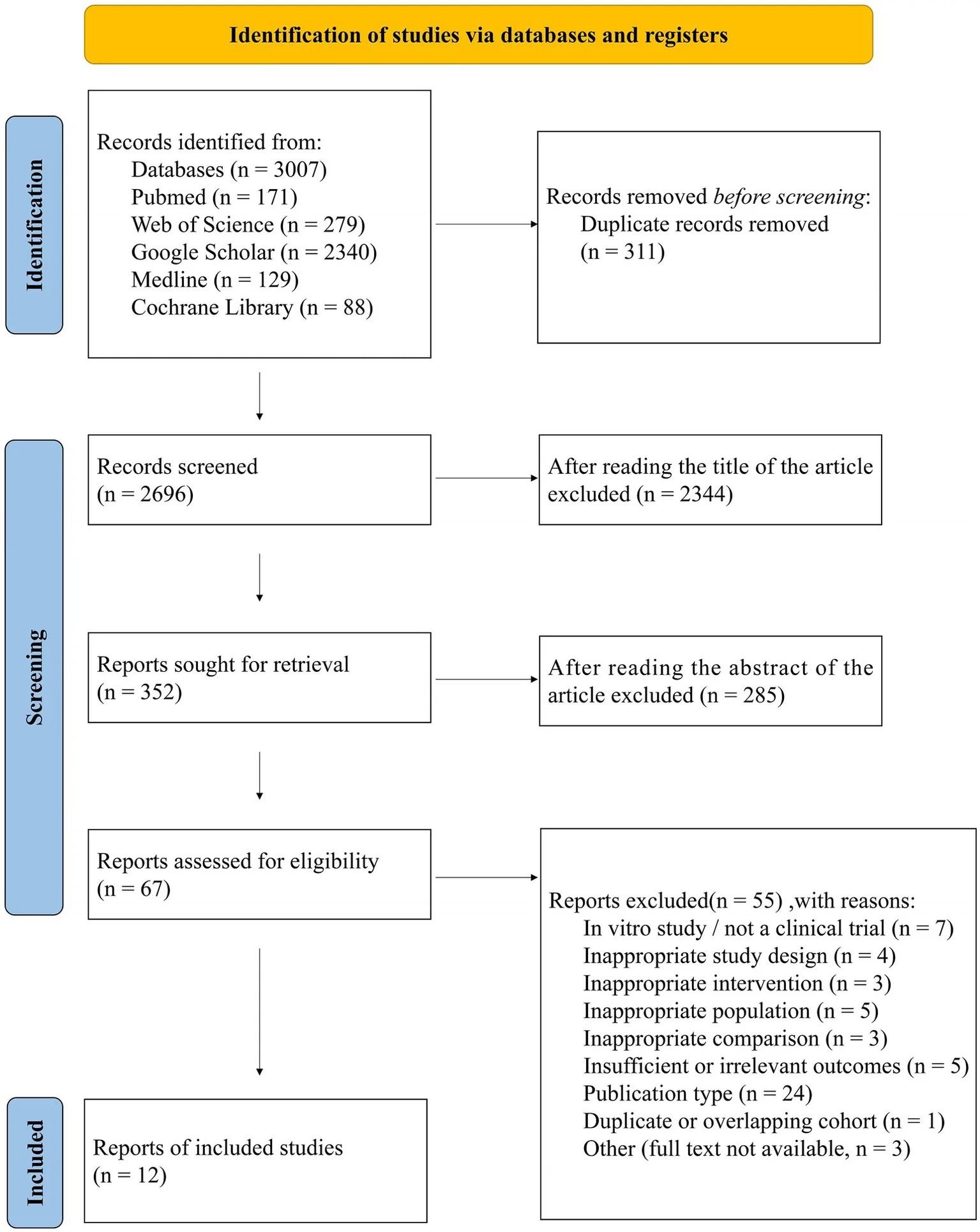
Flow diagram of identified citation and included studies.

Data were extracted using a pre-designed, standardized form. Extracted information included: first author, publication year, country, study design, sample characteristics, intervention and comparator details, follow-up duration, microbial analysis methodology, and all available data regarding changes in the abundance of the target taxa. *Bifidobacterium* was assessed at the genus level and *F. prausnitzii* at the species level, consistent with the reporting in the primary studies.

### 2.5 Determination of effect direction and evidence categorization

Given high methodological heterogeneity in microbial profiling techniques, data reporting formats, and statistical analyses, we used a direction-of-effect and consistency-based synthesis instead of traditional meta-analysis, which pools effect sizes (So et al., 2022; Campbell et al., 2020). The direction of effect (increase [↑], decrease [↓], or no significant change [→]) was determined according to the following pre-specified hierarchical rules (in descending order of strength).

*Rule 1 (Direct between-group comparison)*: Priority was given to the reported *p*-value for a direct comparison between the low-FODMAP diet group and the control group at the endpoint (significance level *α* = 0.05).

*Rule 2 (Author’s explicit statement)*: If no *p*-value was reported, but the authors explicitly stated a statistically significant difference or no difference between groups in the text or figure/table legends, this statement was adopted.

*Rule 3 [LEfSe (Linear Discriminant Analysis Effect Size) between-group enrichment analysis]*: If a study only reported results from a LEfSe analysis, a tax on significantly enriched in the low-FODMAP diet group was categorized as “relatively increased,” and vice versa.

*Rule 4 (Inference based on within-group comparisons)*: For studies providing only within-group pre-post comparison data, an effect was cautiously inferred if a significant change occurred in the low-FODMAP diet group but not in the control group.

*Rule 5 (Handling of multi-group studies)*: For studies with more than two groups, priority was given to *p*-values from direct pairwise comparisons between the target groups. If only a non-significant result from an omnibus test (e.g., ANOVA) was reported, the effect was recorded as “no significant change”.

To enhance transparency, each extracted record was annotated with its corresponding evidence type (Table 1).

**Table 1:** Evidence type and direction of effect for *Bifidobacterium* and *Faecalibacterium prausnitzii (F. prausnitzii)*.

First author (year)	Target genus	Direction of effect	Evidence type	Key supporting information (*p*-value, method, or statement)
*Bifidobacterium*				
McIntosh et al. (2017)	*Bifidobacterium*	↓(Decrease)	Direct_*P*	*p* = 0.041 (uncorrected)
Bennet et al. (2018)	*Bifidobacterium*	↓(Decrease)	Direct_*P*	*p* = 0.020 (adjusted)
Staudacher et al. (2017, 2021)	*Bifidobacterium*	↓(Decrease)	Direct_*P*	*p* = 0.029
Wilson et al. (2020)	*Bifidobacterium*	↓(Decrease)	Direct_*P*	*p* = 0.015
Halmos et al. (2015)	*Bifidobacterium*	↓(Decrease)	Direct_*P*	*p* = 0.028
Staudacher et al. (2012)	*Bifidobacterium*	↓(Decrease)	Direct_*P*	*p* < 0.001
Hustoft et al. (2016)	*Bifidobacterium*	↓(Decrease)	Direct_*P*	*p* = 0.009
Staudacher et al. (2016)	*Bifidobacterium*	↓(Decrease)	Direct_*P*	*p* = 0.028
Zhang et al. (2021)	*Bifidobacterium*	↓(Decrease)	LEfSe_Enriched	Enriched in TDA group (LDA > 0)
Rej et al. (2022)	*Bifidobacterium*	→(No change)	Inferred	Within-group *p* = 0.160
Bin et al. (2018)	*Bifidobacterium*	→(No change)	Direct_*P*	*p* = 0.492 (absolute abundance)
*F. prausnitzii*				
Hustoft et al. (2016)	*F. prausnitzii*	↓(Decrease)	Direct_*P*	*p* = 0.030
Ankersen et al. (2021)	*F. prausnitzii*	↓(Decrease)	Author_Statement	restriction phase: Author explicitly stated reduction reintroduction phase: Increase *p* < 0.05
Wilson et al. (2020)	*F. prausnitzii*	→(No change)	Multiple-group Comparisons	*p* = 0.897 (three-group comparison)
Halmos et al. (2015)	*F. prausnitzii*	→(No change)	Direct_*P*	*p* = 0.108
Staudacher et al. (2012)	*F. prausnitzii*	→(No change)	Direct_*P*	*p* = 0.160
Rej et al. (2022)	*F. prausnitzii*	→(No change)	Inferred	Within-group *p* = 0.740
Zhang et al. (2021)	*F. prausnitzii*	↑(Increase)	LEfSe_Enriched	Enriched in LFD group (LDA < 0)

Direct_P, Direct between-group *p*-value; LEfSe, Linear Discriminant Analysis Effect Size; LDA, Linear Discriminant Analysis score; TDA, Traditional Dietary Advice; LFD, Low-FODMAP Diet.

### 2.6 Statistical analysis (consistency analysis)

For each target taxa, a vote-counting method was first used to describe the distribution of effect directions (increase, decrease, no change) across all included studies. Subsequently, a sign test was applied to assess whether a consistent directional trend existed. Records indicating “no significant change” were treated as not supporting either direction. The null hypothesis was that the probabilities of increase and decrease were equal (50% each). A *p*-value <0.05 (one-tailed) was considered statistically significant, indicating a consistent direction of change. All statistical analyses were performed using SPSS 19.0.0.

### 2.7 Risk of bias assessment and sensitivity analysis

The Cochrane Risk of Bias tool for randomized trials was used to assess the methodological quality of the included RCTs (Barker et al., 2023). To test the robustness of the primary findings, we conducted several sensitivity analyses. First, we repeated the consistency analysis including only studies with the “direct between-group comparison” evidence type, to verify whether the conclusions remained valid under the highest strength of evidence. Second, we stratified the analysis by study design (parallel vs. crossover), by IBS subtype (studies that explicitly excluded constipation-predominant IBS vs. those that included this subtype), by geographic region (Asia vs. Europe/North America), and by control group type (non-high-FODMAP controls vs. high-FODMAP controls), to explore whether the results varied across these characteristics.

## 3 Results

### 3.1 Search results and study selection

The systematic search across five electronic databases (PubMed, Web of Science, Google Scholar, Medline, and the Cochrane Library) initially yielded 3,007 records. After the removal of 311 duplicates, 2,696 unique records underwent title and abstract screening. This process excluded 2,344 records that were clearly irrelevant to the research question. Subsequently, the full texts of the remaining 352 articles were retrieved and assessed for eligibility. A total of 285 articles were excluded after abstract screening, leaving 67 articles for full-text assessment. Of these, a further 55 articles were excluded after full-text review. The primary reasons for exclusion at the full-text stage were: *in vitro* study or not a clinical trial (*n* = 7), inappropriate study design (*n* = 4), inappropriate intervention (*n* = 3), inappropriate population (*n* = 5), inappropriate comparison (*n* = 3), insufficient or irrelevant outcomes (*n* = 5), publication type (*n* = 24), duplicate or overlapping cohort (*n* = 1), and full text not available (*n* = 3).

Ultimately, 12 randomized controlled trials, meeting all pre-defined eligibility criteria, were included in the analysis. The detailed study selection process was illustrated in the PRISMA flow diagram (Figure 1).

### 3.2 Characteristics of included studies

The key characteristics of the 12 included RCTs are summarized in Table 2. The studies were published between 2012 and 2022 and conducted across eight countries (United Kingdom [*n* = 4], China [*n* = 2], Australia, Canada, Denmark, Norway, Sweden, and the United States). The total sample size across all studies was 606 patients with IBS. Sample sizes in individual trials ranged from 20 to 107 participants. All studies enrolled adult IBS patients diagnosed using Rome III or IV criteria. 8 of the 12 studies included only mixed or diarrhea-predominant subtypes, i.e., IBS-D, IBS-M, or IBS-U (Rej et al., 2022; Wilson et al., 2020; Staudacher et al., 2017; Staudacher et al., 2021; Staudacher et al., 2012; Zhang et al., 2021; Hustoft et al., 2016; Ankersen et al., 2021; Bin et al., 2018), while the remaining 4 studies included at least one of these subtypes alongside constipation‑predominant IBS, i.e., IBS‑C (Bennet et al., 2018; McIntosh et al., 2017; Halmos et al., 2015; Staudacher et al., 2016).

**Table 2:** Characteristics of included studies examining the low-FODMAP diet’s effects on gut microbiota in IBS patients.

Study (reference), country	Patients (*n*; age range or Mean age; % Male)	IBS diagnosis (Rome criteria)	Interventions	Adherence	Trial design; duration (days)	Assessment phase	Reported blinding	Microbiome analysis method	Key microbiome finding (s)
McIntosh et al. (2017) Canada	27; 24–83 yr.; 14%	Rome III (IBS-C/D/M/U)	E: Low-FODMAP diet (dietary advice) C: High-FODMAP diet (dietary advice)	Unclear	Parallel; 21	Restriction phase	Single	16S rRNA gene sequencing	↓*Bifidobacterium* (*p* = 0.041)
Bennet et al. (2018) Sweden	61; 18–69 yr.; 19%	Rome III (IBS-C/D/non-Cnon-D)	E: Low-FODMAP diet (dietary advice) C: Traditional IBS diet (NICE/BDA guidelines)	Unclear	Parallel; 28	Restriction phase	Single	GA-map™ Dysbiosis Test	↓*Bifidobacterium* (*p* = 0.020)
Staudacher et al. (2017); Staudacher et al. (2021) United Kingdom	40; 34 yr. (mean); 31%	Rome III (IBS-D/M/U; no C)	E: Low-FODMAP diet + Placebo (dietary advice) C: Sham diet + Placebo (dietary advice)	100%	Parallel; 28	Restriction phase	Single	qPCR; 16S rRNA gene sequencing	↓*Bifidobacterium* (*p* = 0.029)
Wilson et al. (2020) United Kingdom	42; 35 yr. (mean); 44%	Rome III (IBS-D/M/U; no C)	E: Low-FODMAP diet + Placebo (dietary advice) C: Sham diet + Placebo (dietary advice)	91–95%	Parallel; 28	Restriction phase	Single	16S rRNA gene sequencing	↓*Bifidobacterium* (*p* = 0.015) →*F. prausnitzii* (*p* = 0.897)
Halmos et al. (2015) Australia	27; 41 yr. (mean); 22%	Rome III (IBS-C/D/M/U)	E: Low-FODMAP diet (controlled feeding) C: Typical Australian diet (controlled feeding)	80%	Crossover; 21	Restriction phase	Single	DGGE; qPCR	↓*Bifidobacterium* (*p* = 0.028) →*F. prausnitzii* (*p* = 0.108)
Rej et al. (2022) United Kingdom	35; 38 yr. (mean); 26%	Rome IV (IBS-D/M; no C)	E: Low-FODMAP diet (dietary advice) C: Traditional dietary advice (NICE/BDA guidelines)	Unclear	Parallel; 28	Restriction phase	Unblinded	GA-map™ Dysbiosis Test	→*Bifidobacterium* (*p* = 0.160) →*F. prausnitzii* (*p* = 0.740)
Staudacher et al. (2012) United Kingdom	35; 35 yr. (mean); 37%	Rome III (IBS-D/M/U; no C)	E: Low-fermentable carbohydrate diet (dietary advice) C: Habitual diet (dietary advice)	Unclear	Parallel; 28	Restriction phase	Unblinded	FISH	↓*Bifidobacterium* (*p* < 0.001) →*F. prausnitzii* (*p* = 0.160)
Zhang et al. (2021) China	86; 44 yr. (mean); 53%	Rome III (IBS-D only)	E: Low-FODMAP diet (dietary advice) C: Traditional dietary advice (NICE/BDA guidelines)	78%	Parallel; 21	Restriction phase	Unblinded	16S rRNA gene sequencing	↓*Bifidobacterium* (LEfSe) ↑*F. prausnitzii* (LEfSe)
Hustoft et al. (2016) Norway	20;18–52 yr.; 25%	Rome III (IBS-D/M; no C)	E: Low-FODMAP diet period C: High-FOS supplement period (crossover)	Unclear	Crossover; 63 (3-wk LFD + 10-d FOS)	Restriction phase	Double (outcome assessor/supplement)	GA-map™ dysbiosis test; 16S rRNA gene sequencing	↓*Bifidobacterium* (*p* = 0.009) ↓*F. prausnitzii* (*p* = 0.030)
Staudacher et al. (2016) United States	95; NR; NR	Rome III (IBS-C/D/M/U)	E: Low-FODMAP diet (dietary advice) C: Sham diet (dietary advice)	Unclear	Parallel; 28	Restriction phase	Unclear	qPCR	↓*Bifidobacterium* (*p* = 0.028)
Bin et al. (2018) China	107; NR; NR	Rome III (IBS-D; no C)	E: Low-FODMAP diet (dietary advice) C: Normal diet (dietary advice)	Unclear	Parallel; 28	Restriction phase	Double (outcome assessor)	Absolute quantitative count	→*Bifidobacterium* (*p* = 0.492)
Ankersen et al. (2021) Denmark	31; 44 yr. (mean); 29%	Rome III (IBS-D/M; no C)	E: Low-FODMAP diet (Web-app guidance) C: (Crossover design; Baseline/Habitual diet as control)	Unclear	Crossover; 28	Restriction phase and reintroduction phase	Unblinded	Shotgun metagenomic sequencing	↓*F. prausnitzii* (author’s statement) ↑*F. prausnitzii* (*p* < 0.05) (reintroduction phase)

C, control group; DGGE, Denaturing Gradient Gel Electrophoresis; E, experimental group; FISH, Fluorescence in situ Hybridization; FOS, fructo-oligosaccharides; IBS, irritable bowel syndrome; IBS-C/D/M/U, constipation−/diarrhea−/mixed−/unsubtyped-predominant IBS; LFD, low-FODMAP diet; N/A, not applicable; NICE/BDA, National Institute for Health and Care Excellence/British Dietetic Association; NR, not reported; qPCR, quantitative polymerase chain reaction; rRNA, ribosomal RNA; yr, years.

Symbols: ↓, significant decrease; ↑, significant increase; →, no significant change.

The low-FODMAP diet intervention was primarily delivered through individualized dietary advice [10/12 studies (Rej et al., 2022; Staudacher et al., 2017; Hustoft et al., 2016; Halmos et al., 2015; Staudacher et al., 2021; Zhang et al., 2021; Staudacher et al., 2012; Staudacher et al., 2016; Bennet et al., 2018; McIntosh et al., 2017; Bin et al., 2018)] and lasted 3 to 4 weeks in most trials. Comparator diets included sham/placebo diets, habitual diets, high-FODMAP diets, or traditional dietary advice (e.g., NICE/BDA guidelines). Data on adherence were reported in 4 of the 12 included RCTs: Halmos et al. (2015) reported 80% adherence; Staudacher et al. (2021) and Staudacher et al. (2017) reported complete (100%) adherence; Wilson et al. (2020) reported 91–95%; and Zhang et al. (2021) reported 78% adherence. No explicit adherence data were provided by the remaining eight studies.

The microbiome was analyzed using a variety of techniques, including 16S rRNA gene sequencing, qPCR, GA-map Dysbiosis Test, FISH, and shotgun metagenomic sequencing. Among these, 16S rRNA gene sequencing was the most commonly used method [5/12 studies (Wilson et al., 2020; Staudacher et al., 2017; Staudacher et al., 2021; Zhang et al., 2021; Hustoft et al., 2016; McIntosh et al., 2017)]. As pre-specified in our analytical focus, 11 of the 12 studies reported data on *Bifidobacterium* abundance (McIntosh et al., 2017; Bennet et al., 2018; Staudacher et al., 2021; Staudacher et al., 2017; Wilson et al., 2020; Halmos et al., 2015; Rej et al., 2022; Staudacher et al., 2012; Zhang et al., 2021; Hustoft et al., 2016; Staudacher et al., 2016; Bin et al., 2018), and seven studies reported data on *F. prausnitzii* (Rej et al., 2022; Halmos et al., 2015; Staudacher et al., 2012; Zhang et al., 2021; Hustoft et al., 2016; Wilson et al., 2020; Ankersen et al., 2021).

### 3.3 Results of the direction of effect and consistency analysis

#### 3.3.1 Bifidobacterium

A total of 11 studies reported on the effects of a low-FODMAP diet on the abundance of intestinal *Bifidobacterium* in IBS patients. The direction of effect analysis revealed a predominant trend of reduction (Table 3). 9 studies (81.8%) reported a significant decrease in *Bifidobacterium* abundance (McIntosh et al., 2017; Bennet et al., 2018; Staudacher et al., 2017; Staudacher et al., 2021; Wilson et al., 2020; Halmos et al., 2015; Staudacher et al., 2012; Hustoft et al., 2016; Staudacher et al., 2016; Zhang et al., 2021), while 2 studies reported no significant change (Rej et al., 2022; Bin et al., 2018). No study reported an increase. To evaluate the statistical significance of this dominant “decrease” trend, a sign test (binomial test, test proportion = 0.5) was performed. The result indicated that the consistency of a reduction in *Bifidobacterium* following a low-FODMAP diet was statistically significant (*p* = 0.033).

**Table 3:** Direction of effect of a low-FODMAP diet on the abundance of *Bifidobacterium*.

Direction of effect	Number of studies (*n*)	Proportion (%)
Decrease (↓)	9	81.8
No change (→)	2	18.2
Increase (↑)	0	0.0
Total	11	100.0

*p* = 0.033 (one-tailed sign test, comparing the number of studies reporting a decrease versus those reporting no change or an increase).

#### 3.3.2 Faecalibacterium prausnitzii

7 studies reported data on *F. prausnitzii* abundance. The distribution of effect directions was heterogeneous (Table 4): two studies reported a significant decrease (Hustoft et al., 2016; Ankersen et al., 2021), 4 reported no significant change (Rej et al., 2022; Wilson et al., 2020; Staudacher et al., 2012; Halmos et al., 2015), and 1 reported a significant increase (Zhang et al., 2021). The sign test (binomial test, test proportion = 0.5) showed that the current evidence does not support a consistent directional change (*p* = 0.227 for a “decrease” trend).

**Table 4:** Direction of effect of a low-FODMAP diet on the abundance of *F. prausnitzii*.

Direction of effect	Number of studies (*n*)	Proportion (%)
Decrease (↓)	2	28.6
No change (→)	4	57.1
Increase (↑)	1	14.3
Total	7	100.0

*p* = 0.227 (one-tailed sign test, comparing the number of studies reporting a decrease versus those reporting no change or an increase).

#### 3.3.3 Sensitivity analysis

To test the robustness of the primary findings, a pre-specified sensitivity analysis was conducted, including only studies with the “Direct between-group comparison *p*-value” evidence type (Table 5). For *Bifidobacterium*, among the 9 eligible studies, 8 (88.9%) reported a significant decrease (Wilson et al., 2020; Staudacher et al., 2017; Staudacher et al., 2021; Staudacher et al., 2012; Hustoft et al., 2016; Staudacher et al., 2016; McIntosh et al., 2017; Bennet et al., 2018; Halmos et al., 2015), and 1 reported no change (Bin et al., 2018). The sign test confirmed that the consistency of the reduction was highly statistically significant (*p* = 0.020). For *F. prausnitzii*, only three studies provided direct comparison evidence [two reporting no change (Staudacher et al., 2012; Halmos et al., 2015) and 1 reporting a decrease (Hustoft et al., 2016)], which was insufficient for a meaningful statistical test. The sensitivity analysis supported the conclusion regarding *Bifidobacterium* from the primary analysis and underscored the current lack of high-quality direct comparative evidence for *F. prausnitzii*.

**Table 5:** Sensitivity analysis: direction of effect for *Bifidobacterium* and *F. prausnitzii* stratified by study characteristics.

Stratification	*Bifidobacterium*					*F. prausnitzii*				
	Total (*n*)	Decrease [*n* (%)]	No change [*n* (%)]	Increase [*n* (%)]	Sign test (*p*)	Total (*n*)	Decrease [*n* (%)]	No change [*n* (%)]	Increase [*n* (%)]	Sign test (*p*)
Evidence type										
Direct_*P* only	9	8 (88.9%)	1 (11.1%)	0 (0.0%)	0.020	3	1 (33.3%)	2 (66.7%)	0 (0.0%)	N/A^a^
Study design										
Parallel	9	7 (77.8%)	2 (22.2%)	0 (0.0%)	0.090	4	0 (0.0%)	3 (75.0%)	1 (25.0%)	N/A^a^
Crossover	2	2 (100.0%)	0 (0.0%)	0 (0.0%)	N/A^a^	3	2 (66.7%)	1 (33.3%)	0 (0.0%)	0.500§
IBS subtype										
Studies excluding IBS-C	7	5 (71.4%)	2 (28.6%)	0 (0.0%)	0.226	6	2 (33.3%)	3 (50.0%)	1 (16.7%)	0.344§
Studies including IBS-C	4	4 (100.0%)	0 (0.0%)	0 (0.0%)	N/A^a^	1	0 (0.0%)	1 (100.0%)	0 (0.0%)	N/A^a^
Region										
Asia	2	1 (50.0%)	1 (50.0%)	0 (0.0%)	N/A^a^	1	0 (0.0%)	0 (0.0%)	1 (100.0%)	N/A^a^
Europe/North America	9	8 (88.9%)	1 (11.1%)	0 (0.0%)	0.020	6	2 (33.3%)	4 (66.7%)	0 (0.0%)	0.344§
Control group										
Non-high-FODMAP controls	9	7 (77.8%)	2 (22.2%)	0 (0.0%)	0.090	6	1	4	1	0.110§
High-FODMAP controls	2	2 (100.0%)	0 (0.0%)	0 (0.0%)	N/A^a^	1	1(100.0%)	0(0.0%)	0(0.0%)	N/A^a^

Direct_P, Direct between-group *p*-value; IBS, irritable bowel syndrome; IBS-C, constipation-predominant IBS.

^a^N/A: Not applicable due to an insufficient number of studies for a meaningful statistical test.

§Sign test performed comparing decrease vs. non-decrease (no change or increase).

Non-high-FODMAP Controls: Control groups comprised Traditional IBS diet, Habitual diet, Sham diet, or Typical Australian diet.

Some subgroup analyses have limited sample sizes; results should be interpreted with caution.

We further explored the robustness by stratifying the analysis according to study characteristics (Table 5). When grouped by study design, the trend toward *Bifidobacterium* reduction was observed in both parallel and crossover designs. Among the 9 parallel-designed studies, 7 reported a decrease (Staudacher et al., 2017; Wilson et al., 2020; Staudacher et al., 2021; Staudacher et al., 2012; Zhang et al., 2021; Staudacher et al., 2016; Bennet et al., 2018; McIntosh et al., 2017), and 2 reported no change (Rej et al., 2022; Bin et al., 2018), with *p* = 0.090. Among the 2 crossover-designed studies (Hustoft et al., 2016; Halmos et al., 2015), both reported a decrease. For *F. prausnitzii*, none of the 4 parallel-designed studies reported a decrease (Wilson et al., 2020; Rej et al., 2022; Staudacher et al., 2012; Zhang et al., 2021). Among the 3 crossover-designed studies, 2 reported a decrease (Hustoft et al., 2016; Ankersen et al., 2021) and 1 reported no change (Halmos et al., 2015), with *p* = 0.500.

When stratified by IBS subtype, the reduction in *Bifidobacterium* was observed in 5 out of 7 studies that excluded constipation-predominant IBS, with *p* = 0.226 for this subgroup (Staudacher et al., 2017; Wilson et al., 2020; Rej et al., 2022; Staudacher et al., 2021; Staudacher et al., 2012; Zhang et al., 2021; Hustoft et al., 2016; Bin et al., 2018) and the reduction was also observed in all 4 studies that included this subtype (Staudacher et al., 2016; Bennet et al., 2018; McIntosh et al., 2017; Halmos et al., 2015). For *F. prausnitzii*, among the 6 studies that excluded IBS-C, 2 reported a decrease (Hustoft et al., 2016; Ankersen et al., 2021), while 4 reported no change or increase (Wilson et al., 2020; Rej et al., 2022; Staudacher et al., 2012; Zhang et al., 2021), with *p* = 0.344 for this subgroup; only one study included IBS-C (Halmos et al., 2015), which reported no change.

When stratified by geographic region, the reduction in *Bifidobacterium* was observed in 8 out of 9 European/North American studies, with *p* = 0.020 (Staudacher et al., 2017; Wilson et al., 2020; Rej et al., 2022; Staudacher et al., 2021; Staudacher et al., 2012; Hustoft et al., 2016; Staudacher et al., 2016; Bennet et al., 2018; McIntosh et al., 2017; Halmos et al., 2015), whereas only 1 of the 2 Asian studies showed a reduction (Zhang et al., 2021; Bin et al., 2018). For *F. prausnitzii*, only 2 out of 6 studies in the European/North American subgroup reported a decrease (Wilson et al., 2020; Rej et al., 2022; Staudacher et al., 2012; Hustoft et al., 2016; Ankersen et al., 2021; Halmos et al., 2015), indicating a non-significant trend toward no change (*p* = 0.344), while the Asian subgroup contained only one study (Zhang et al., 2021).

We also performed a sensitivity analysis based on whether the control group received a high-FODMAP diet. For *Bifidobacterium*, 7 out of 9 studies using a control diet that was not high in FODMAPs reported a decrease (Bennet et al., 2018; Staudacher et al., 2021; Staudacher et al., 2017; Wilson et al., 2020; Staudacher et al., 2012; Staudacher et al., 2016; Zhang et al., 2021; Halmos et al., 2015), with *p* = 0.090. The remaining 2 studies, both of which used a high-FODMAP control, reported a decrease (McIntosh et al., 2017; Hustoft et al., 2016). For *F. prausnitzii*, among the 6 studies using a control diet that was not high in FODMAPs, only 1 reported a decrease (Ankersen et al., 2021), while 4 reported no change (Wilson et al., 2020; Halmos et al., 2015; Staudacher et al., 2012; Rej et al., 2022) and 1 reported an increase (Zhang et al., 2021), with *p* = 0.110 for this subgroup. The only study that used a high-FODMAP control reported a decrease (Hustoft et al., 2016).

These stratified analyses should be interpreted with caution due to the limited sample sizes in some subgroups.

### 3.4 Microbial changes during food reintroduction

Among the 12 enrolled RCTs, most (*n* = 11) evaluated the intestinal microbiota exclusively during the strict restriction period; only one study (Ankersen et al., 2021) concurrently assessed gut microbiota dynamics during a 4-week strict low-FODMAP diet and the following high-FODMAP food reintroduction phase (Table 2). In this study, *F. prausnitzii*, which was significantly reduced during the strict low-FODMAP diet phase, recovered after high-FODMAP foods were reintroduced. Notably, the data on *Bifidobacterium* was not reported in this trial. This observation suggested that some low-FODMAP diet-induced microbial changes may be reversible.

### 3.5 Other microbiome-related outcomes

Only 4 studies reported α-diversity, 4 reported β-diversity, 5 reported SCFAs, and 5 reported overall microbial composition. The summary is provided in Supplementary Table S1.

For *α*-diversity (4 studies), 3 found no significant differences between groups (Wilson et al., 2020; McIntosh et al., 2017; Staudacher et al., 2021; Staudacher et al., 2017), while 1 observed an increase following the low-FODMAP diet compared with the control (Zhang et al., 2021).

For β-diversity (4 studies), none reported significant changes (McIntosh et al., 2017; Staudacher et al., 2021; Staudacher et al., 2017; Zhang et al., 2021; Wilson et al., 2020).

For SCFAs (5 studies), 3 found no intergroup differences (Halmos et al., 2014; Wilson et al., 2020; Staudacher et al., 2012); among the 2 studies reporting within-group changes, 1 showed a significant reduction in total SCFAs (Hustoft et al., 2016), while 1 reported a decrease in butyrate but increases in isobutyrate and isovalerate, with no changes in acetate, propionate, or valerate (Zhang et al., 2021).

For overall microbial composition (5 studies), 4 reported no differences (McIntosh et al., 2017; Wilson et al., 2020; Staudacher et al., 2012; Zhang et al., 2021), and 1 reported a reduction in total bacterial abundance (Halmos et al., 2014).

### 3.6 Risk of bias and GRADE

The methodological quality of the 12 included randomized controlled trials was assessed using the Cochrane Risk of Bias tool version 2.0 (Principi et al., 2018), which evaluated bias across five domains specifically for the pre-defined outcome: changes in the abundance of *Bifidobacterium* and *F. prausnitzii*.

In the randomization process (D1), nine studies were judged to have some concerns primarily due to insufficient detail on allocation concealment (Rej et al., 2022; Staudacher et al., 2017; Staudacher et al., 2021; Hustoft et al., 2016; Staudacher et al., 2016; Ankersen et al., 2021; McIntosh et al., 2017; Bennet et al., 2018; Bin et al., 2018; Halmos et al., 2015), while three studies were rated as low risk (Wilson et al., 2020; Staudacher et al., 2012; Zhang et al., 2021).

For deviations from intended interventions (D2), all 12 studies were judged to be at high risk of bias. This judgment arose from the inherent nature of dietary interventions: regardless of how studies described their blinding procedures (e.g., “double-blind”), participants could not be genuinely blinded to the allocated diet (low-FODMAP vs. control) because they consume and experience the food. This knowledge may lead to differential adherence or co-interventions.

In the domain of missing outcome data (D3), the majority of studies (*n* = 9) were rated as low risk due to low and balanced dropout rates (Rej et al., 2022; Wilson et al., 2020; Staudacher et al., 2017; Staudacher et al., 2021; Staudacher et al., 2012; Hustoft et al., 2016; Staudacher et al., 2016; McIntosh et al., 2017; Bennet et al., 2018; Halmos et al., 2015). One study (Ankersen et al., 2021) was rated as high risk due to a high attrition rate (35%), and two others had some concerns (Zhang et al., 2021; Bin et al., 2018).

Measurement of the outcome (D4) was consistently rated as low risk across all studies, as the gut microbiome analysis (16S rRNA sequencing, qPCR, etc.) constituted an objective laboratory measurement unlikely to be influenced by knowledge of the intervention.

Regarding the selection of the reported result (D5), 10 studies with prospective registration that explicitly mentioned microbiome analysis were rated as low risk. Three studies were rated as having some concerns: one had registration but did not pre-specify microbiome outcomes (Bennet et al., 2018), and two lacked prospective registration information (Hustoft et al., 2016; Bin et al., 2018).

Consequently, following the RoB 2.0 algorithm, the overall risk of bias was judged as high for all 12 studies, driven by the universal high risk in domain D2. However, given that the core microbiome outcomes were objective laboratory measures, the direct impact of this performance bias on our findings was likely mitigated. A visual summary is provided in Figure 2.

**Figure 2:**
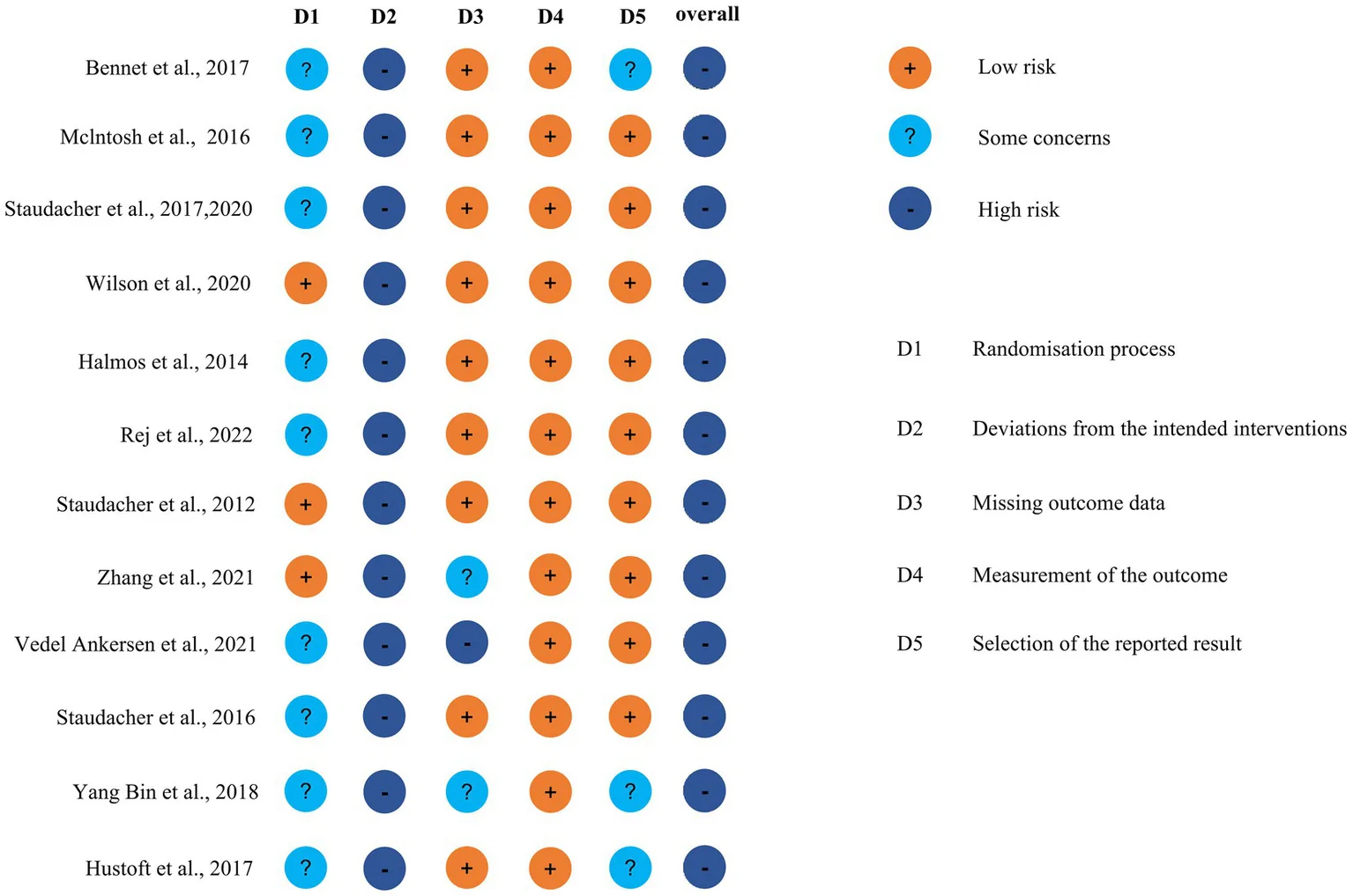
Summary of risk of bias assessments for included trials via Cochrane Risk of Bias 2.0.

## 4 Discussion

### 4.1 Main findings

This review used a method that focuses on the direction of change to summarize evidence on how a low FODMAP diet affects two important gut bacteria in IBS patients. The main finding was that the low-FODMAP diet significantly reduced *Bifidobacterium* abundance in IBS patients, while evidence for *F. prausnitzii* was mixed. This directional trend was consistent across sensitivity and subgroup analyses.

### 4.2 Interpretation and clinical implications

The low-FODMAP diet’s effect on *Bifidobacterium* itself was consistent across most studies. This meant that while patients start in different places and end up with different results, the diet’s direct action on this particular bacterium is more predictable. In contrast, changes in *F. prausnitzii* were inconsistent across studies, suggesting that this species is less specifically regulated by FODMAP restriction and may be modulated by broader individual or environmental confounders.

Importantly, the present results highlight a notable clinical paradox of the low-FODMAP diet: although this dietary intervention effectively alleviates IBS symptoms, its restrictive nature consistently depletes beneficial *Bifidobacterium* populations. The beneficial effects of the low-FODMAP diet on IBS symptoms are primarily attributed to reduced intestinal gas production and luminal distension through the restriction of fermentable substrates (Staudacher et al., 2017). However, as FODMAPs serve as important nutrient sources for *Bifidobacterium*, the reduction in substrate availability inevitably leads to a decrease in the abundance of this genus (McIntosh et al., 2017; Pokusaeva et al., 2011). During short-term strict restriction, the symptom improvement appears to outweigh the potential negative impact of *Bifidobacterium* reduction. Nevertheless, IBS patients already have a lower baseline *Bifidobacterium* level than healthy individuals, and the sustained strict restriction may cause further depletion, potentially impairing gut barrier integrity and anti-inflammatory capacity (Roberfroid et al., 2010; Bootz-Maoz et al., 2022).

These findings have practical implications for the clinical management of patients with IBS. The low-FODMAP diet is typically implemented as a stepwise approach: an initial period of strict restriction (usually 4–8 weeks), followed by gradual reintroduction of foods to identify individual triggers (Bellini and Rossi, 2018). Our findings support this structured method. For this reason, the strict restriction phase of the low-FODMAP diet should be limited to the shortest effective duration, followed by gradual reintroduction of FODMAPs. For patients requiring long-term dietary restriction, prebiotic or probiotic supplementation may help restore *Bifidobacterium* abundance and preserve gut homeostasis. For instance, one trial indicated that supplementation with a specific probiotic (VSL#3) may mitigate the diet-induced decline in *Bifidobacterium* abundance (Staudacher et al., 2017). What’s more, future studies could also explore optimized low-FODMAP regimens that preserve symptom control while retaining a carbohydrate fraction that supports beneficial bifidobacteria.

Taken together, these results support evidence-based clinical counselling, enabling clinicians to inform patients about the microbial consequences of the low-FODMAP diet and guide shared, individualised decisions for dietary management.

### 4.3 Comparison with previous reviews

Previous reviews have explored the influence of a low-FODMAP diet on *Bifidobacterium*, yet most rely on non-quantitative descriptive summaries without formal analytical procedures. Yang et al. (2025), van Lanen et al. (2021), and So et al. (2022) all descriptively reported a reduction in *Bifidobacterium* abundance after intervention. While the directional change we observed aligns with these earlier qualitative findings, our study goes beyond simple narrative description by conducting a set of rigorous statistical analyses, which provides formal statistical validation for this decreasing trend. Available evidence for *F. prausnitzii* remains extremely limited. So et al. (2022) reviewed 4 relevant trials but performed no statistical synthesis and identified no uniform trend. Using the rigorous analytical approach, our study similarly found no consistent directional change in *F. prausnitzii*. To our knowledge, no other published reviews have specifically evaluated the effect of a low-FODMAP diet on *F. prausnitzii*.

Another existing controversy in the literature is whether a patient’s baseline gut microbiota can predict dietary response (Bennet et al., 2018; Valeur et al., 2018). This disagreement appears to stem from studies using different inclusion criteria and definitions of treatment success (Valeur et al., 2018). To facilitate cross-study comparisons in future research, it would be helpful if studies adopted unified, well-accepted definitions of therapeutic success and reported detailed patient characteristics.

### 4.4 Potential biological mechanisms

Beyond substrate deprivation, the effect of the low-FODMAP diet on *Bifidobacterium* may involve additional mechanisms. This dietary intervention may disrupt cross-feeding interactions with butyrate-producing bacteria (Belenguer et al., 2006) and shift niche competition toward bacteria with broader carbohydrate utilization capabilities, such as certain Bacteroides species (Crouch et al., 2022). For *F. prausnitzii*, we did not find a consistent effect, which may be attributable to differences in study design, characteristics of included patient populations, and habitual dietary patterns across geographical regions (Zhang et al., 2021; So et al., 2022). A recent study proposed that IBS patients can be stratified into distinct subtypes based on gut microbial profiles, and that the low-FODMAP diet primarily alters the microbiota in a specific “pathogenic-like” subtype (Vervier et al., 2022). This suggests that the proportion of this patient subtype in each study may have contributed to the observed heterogeneity in overall results. Future research should aim to identify these patient subtypes prior to intervention to better understand who benefits most from the diet.

### 4.5 Strengths, limitations, and future perspectives

The major strength of this review lies in the application of systematic and rigorous analytical approaches. Although previous reviews have addressed the effects of a low-FODMAP diet on gut microbiota, they remain largely narrative and rely on selective citations without systematically synthesizing consistent directional trends. By contrast, the methodological advantage of the present review lies in its adoption of direction-of-effect and consistency analyses, which enable robust data synthesis despite substantial between-study heterogeneity, thereby avoiding subjective citation bias. Additionally, we performed sensitivity analyses restricted to direct between-group comparisons and subgroup analyses stratified by study design, IBS subtype, and geographic region. To our knowledge, this is the first systematic review to comprehensively assess the effects of low-FODMAP diets on *Bifidobacterium* and *F. prausnitzii* in IBS patients.

Nevertheless, several limitations of this review should be acknowledged. First, the direction-of-effect and consistency analysis could not quantify the magnitude of alterations in microbial abundance. Second, inherent discrepancies in the study design and methodological protocols of the included RCTs could not be fully eliminated or adjusted for. Third, the reliability and comprehensiveness of our conclusions were constrained by substantial variation in the completeness and quality of data reporting across the original studies. Fourth, owing to the extremely limited number of eligible studies and substantial heterogeneity in outcome reporting, direction-of-effect and consistency analyses were not feasible for community-level microbiota metrics, nor for other bacterial taxa.

Given the methodological heterogeneity and variable reporting quality across existing studies, future research should prioritize establishing more comprehensive protocols for microbiome sampling, sequencing, and reporting, as well as the adoption of standardized and objective metrics for dietary adherence assessment, to improve the reliability and interpretability of primary study results. Additionally, studies should stratify participants by baseline microbiome subtypes, adopt uniform and widely accepted definitions of treatment success, report detailed patient characteristics, and conduct extended follow-up during the reintroduction phase. These efforts will ultimately generate higher-quality evidence, refine the clinical application of the low-FODMAP diet, and enable more personalized dietary strategies for IBS management.

## 5 Conclusion

In conclusion, this review demonstrates that the low-FODMAP diet significantly reduces *Bifidobacterium* abundance in IBS patients, while evidence for *F. prausnitzii* remains inconclusive. These findings may assist clinicians in counselling patients regarding expected microbial changes and support informed dietary decision-making. However, the current evidence is constrained by methodological heterogeneity, variability in data reporting, limited reporting of community-level metrics, and a paucity of data on the reintroduction phase. Future studies should adopt standardised microbiome protocols, objective adherence measures, and extended follow-up through the reintroduction phase, alongside stratification by baseline microbiota subtypes, to generate higher-quality evidence and refine personalised dietary strategies for IBS management.

## Data Availability

The original contributions presented in the study are included in the article/Supplementary material, further inquiries can be directed to the corresponding author.
