# Supplementary material for: Evidence consistency analysis of the effect of a low-FODMAP diet on the abundance of Bifidobacterium and Faecalibacterium prausnitzii in patients with irritable bowel syndrome
Source: Front Microbiol. 2026 Oct 6;17:1948754. doi: 10.3389/fmicb.2026.1948754 (PMC13680177; doi:10.3389/fmicb.2026.1948754)
Supplement: Supplementary file 1 [file Supplementary_file_1.docx]

Supplementary Material

**1. Graphical Abstract**

**2. Practitioner Points**

Ⅰ. Strict adherence to a low-FODMAP diet significantly reduces the abundance of *Bifidobacterium* in IBS patients, whereas evidence on *F. prausnitzii* remains mixed. Therefore, clinicians should be vigilant about the potential risk of gut microbial dysbiosis induced by long-term strict low-FODMAP restriction.

Ⅱ. A standard three-phase low-FODMAP diet—elimination, reintroduction, and personalization—is recommended to avoid prolonged strict restriction, thereby alleviating IBS symptoms while maintaining gut microbial homeostasis.

Ⅲ. For IBS patients requiring long-term maintenance of a low-FODMAP diet, supplementation with probiotics containing *Bifidobacterium* may help maintain gut microbial homeostasis.

Ⅳ. Evidence on gut microbiota changes during the low-FODMAP diet reintroduction phase remains limited. Additionally, microbial responses to the diet may vary across microbiota subtypes. Future studies should stratify patients by baseline microbiota subtypes and focus on the dynamics of gut microbiota during the reintroduction phase, in order to precisely evaluate individual microbial responses to the diet.

Table S1. Summary of α‑diversity, β‑diversity, SCFA, and overall microbial composition outcomes reported in the included studies.

| Stratification | α‑Diversity | |  | β‑Diversity | |  | SCFA | Overall Microbial Composition |
| --- | --- | --- | --- | --- | --- | --- | --- | --- |
|  | Chao 1 | Shannon |  | Bray Curtis | PCoA |  |  |  |
| McIntosh et al. (2017) | No difference  (*P* = 0.401) | **/** |  | No difference | **/** |  | **/** | No difference |
| Bennet et al. (2018) | **/** | **/** |  | / | **/** |  | **/** | **/** |
| Staudacher et al. (2017); Staudacher et al. (2021) | No difference  (*P* = 0.757) | No difference  (*P* = 0.275) |  | No difference  （*P* = 0.575） | **/** |  | **/** | **/** |
| Wilson et al. (2020) | No difference  (*P* = 0.732) | No difference  (*P* = 1.000) |  | No difference  （*P* = 1.000） | **/** |  | No difference (*P* = 0.068)  Transient butyrate reduction at week 1 | No difference  (three-group comparison) |
| Halmos et al. (2015) | **/** | **/** |  | **/** | **/** |  | No difference (*P* = 0.208) | Decrease: total bacterial abundance (*P* < 0.001) |
| Rej et al. (2022) | **/** | **/** |  | **/** | **/** |  | **/** | **/** |
| Staudacher et al. (2012) | **/** | **/** |  | **/** | **/** |  | No difference | No difference |
| Zhang et al. (2021) | **/** | Increase  (*P* = 0.016) |  | **/** | No difference |  | Decrease: n-butyric acid (within-group *P* = 0.045)  Increase: i-butyric and i-valeric acids (within-group *P* = 0.011 and *P* = 0.009)  No difference: Acetic acid, Propionic acid, Valeric acid | No difference |
| Hustoft et al. (2016) | **/** | **/** |  | **/** | **/** |  | Decrease  (within-group *P* = 0.035） | **/** |
| Staudacher et al. (2016) | **/** | **/** |  | **/** | **/** |  | **/** | **/** |
| Bin et al. (2018) | **/** | **/** |  | **/** | **/** |  | **/** | **/** |
| Ankersen et al. (2021) | **/** | **/** |  | **/** | **/** |  | **/** | **/** |

Abbreviations: PCoA, principal coordinates analysis; SCFA, short‑chain fatty acids.

"No difference" indicates no significant between‑group differences in the reported metric.

"within‑group" indicates significant changes from baseline within the low‑FODMAP diet group.

"Transient" indicates a significant change observed at one time point that was not sustained at later time points.

"**/**" indicates that this indicator was not mentioned in the included studies.
